# Supplementary material for: Coupling fission and exit of RAB6 vesicles at Golgi hotspots through kinesin-myosin interactions
Source: Nat Commun. 2017 Nov 1;8:1254. doi: 10.1038/s41467-017-01266-0 (PMC5665954; doi:10.1038/s41467-017-01266-0)
Supplement: Supplementary file 1 — Supplementary Information [file 41467_2017_1266_MOESM1_ESM.pdf]

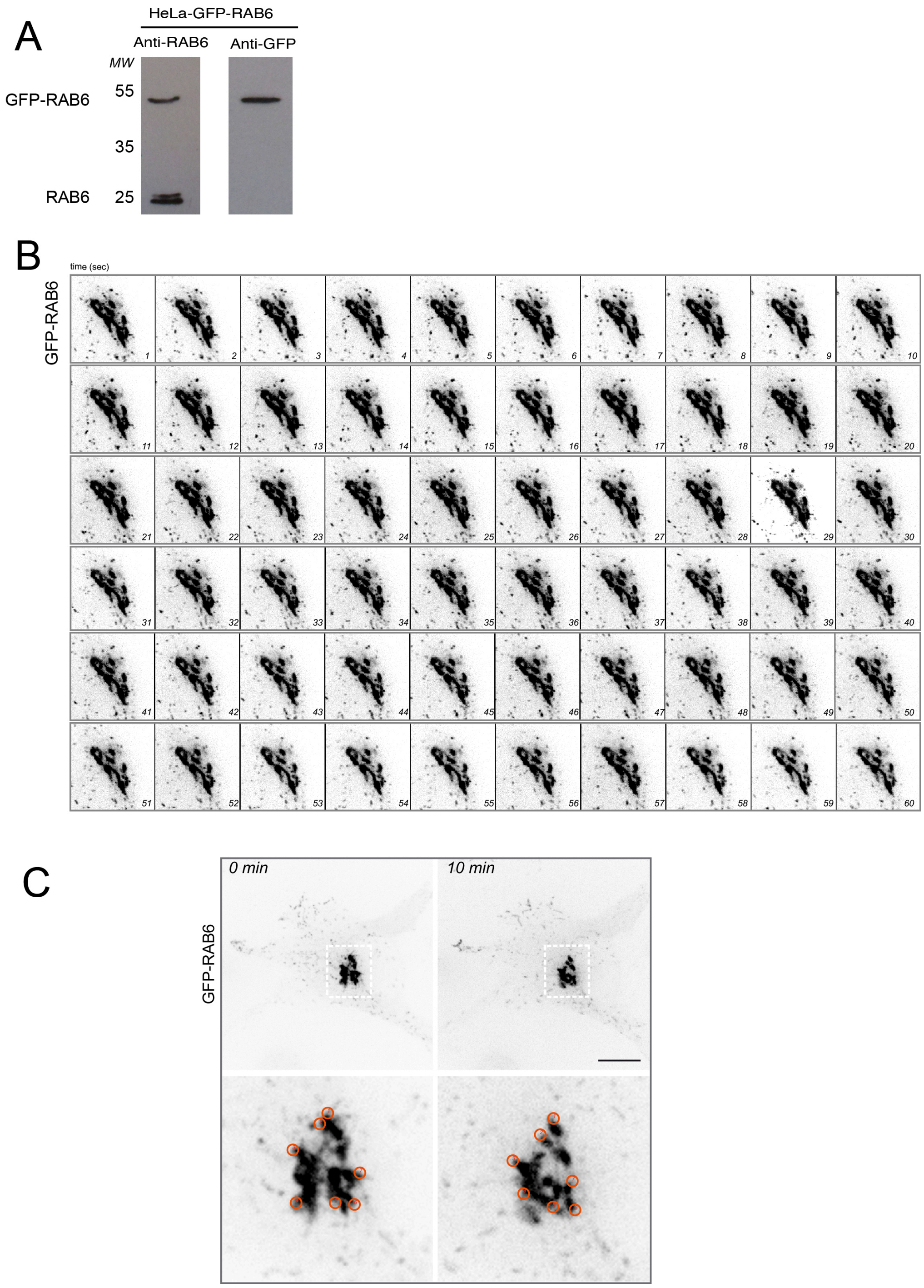

Supplementary Figure 1: GFP-RAB6 is expressed at similar level than endogenous RAB6. Details of the Fission hotspots. Fission hotspots are stable over time.

(A) Western-blot analysis of HeLa cells stably expressing GFP-RAB6. GFP-RAB6 was revealed with a RAB6 and a GFP antibody. *MW*, molecular weight in kDa. **(B)** Time-lapse images corresponding to supplementary movie 1 are displayed. **(C)** GFP-RAB6 HeLa cell was imaged every second for 1 min. Orange circles show location of 7 fission hotspots. The same cell was imaged after 10 min. Orange circles show location of 7 fission hotspots. See corresponding Supplementary movie 2.

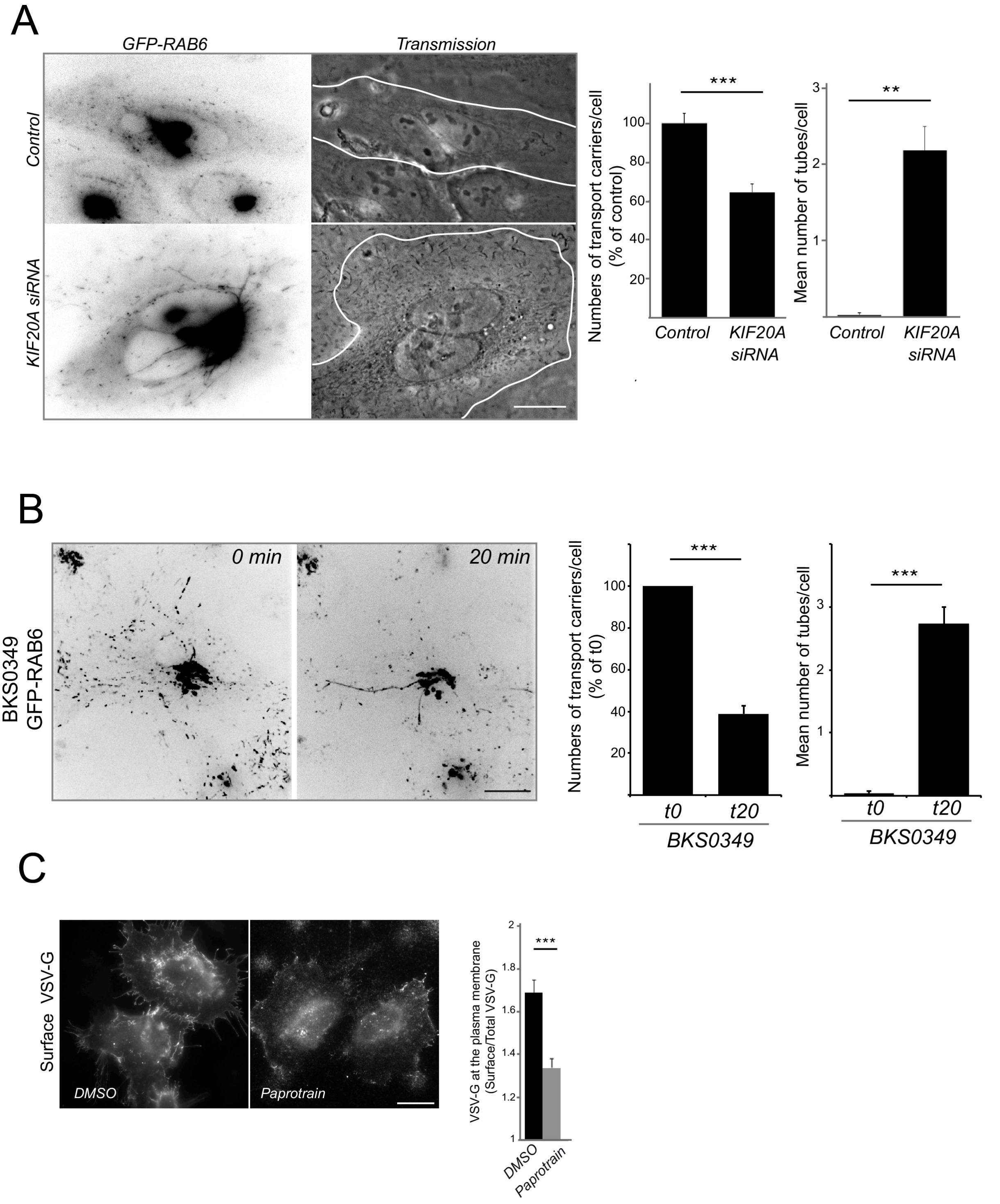
A
GFP-RAB6
Transmission
Control
KIF20A siRNA
Numbers of transport carriers/cell (% of control)
***
Mean number of tubes/cell
**
Control
KIF20A siRNA
B
BKS0349 GFP-RAB6
0 min
20 min
Numbers of transport carriers/cell (% of t0)
***
Mean number of tubes/cell
t0
t20
BKS0349
C
Surface VSV-G
DMSO
Paprotrain
VSV-G at the plasma membrane (Surface/Total VSV-G)
***

Supplementary Figure 2: KIF20A depletion or inhibition leads to an inhibition of fission from the Golgi complex. KIF20A inhibition delays the arrival of VSV-G at the plasma membrane.

(A) Left panel: GFP-RAB6 expressing HeLa cells were imaged by time-lapse microscopy after a 3-day transfection with control condition or KIF20A specific siRNAs. Bi-nucleated cells overexpressing inactive RAB35 mutant which leads to cytokinesis defect [1] were used as the control condition. Indeed, KIF20A is required for cytokinesis and KIF20A depletion leads to binucleated cells [2,3]. Of note, RAB35 has no known function at the Golgi apparatus. Images have been contrasted to allow an easier visualization of the membrane tubes. Transmission images show that cells are bi-nucleated. Right panel: Quantification of the number of transport carriers and of Golgi-connected membrane tubes after KIF20A depletion (n= 16 cells to count the number of tubes or 62-91 squares (16 cells in total) of 100 pixels to count the number of vesicles per cell). *** $p < 10^{-8}$, ** $p = 0.001$ (Student's t-test). Bar, 10 µm. **(B)** Left panel : HeLa cells stably expressing GFP-RAB6 were imaged by time-lapse microscopy, and images of the same cell before (t0) or 20 min (t20) after BKS0349 (1 µM) treatment are presented. BKS0349 was used as a specific inhibitor of KIF20A function [4]. Right panel: Quantification of the number of transport carriers and of Golgi-connected membrane tubes after expression of the different dominant negative mutants. Results are expressed as mean ± SEM (n= 27-59 cells). *** $p < 10^{-10}$ (Student's t-test). Bar, 10 µm. **(C)** KIF20A inhibition delays the arrival of VSV-G at the plasma membrane. HeLa cells expressing VSV-G-GFP tsO45 thermosensitive mutant were incubated overnight at 40°C. Cells were then shifted to 32°C for 1.5 h to allow VSV-G to reach the plasma membrane. Cells stained for VSV-G at the plasma membrane ("surface VSV-G" revealed by an anti-ectodomain antibody, in grey levels) are displayed. VSV-G was first released from 40°C for 30 min to allow its transport to the Golgi, before paprotrain was added. Quantifications of VSV-G at the plasma membrane (surface VSV-G to total VSV-G ratio) 1.5 h after shifting the cells to 32°C (mean ± SEM, n= 35-37 cells). *** $p < 10^{-4}$ (Student's t-test). Similar effects were observed following KIF20A depletion by siRNA (data not show).

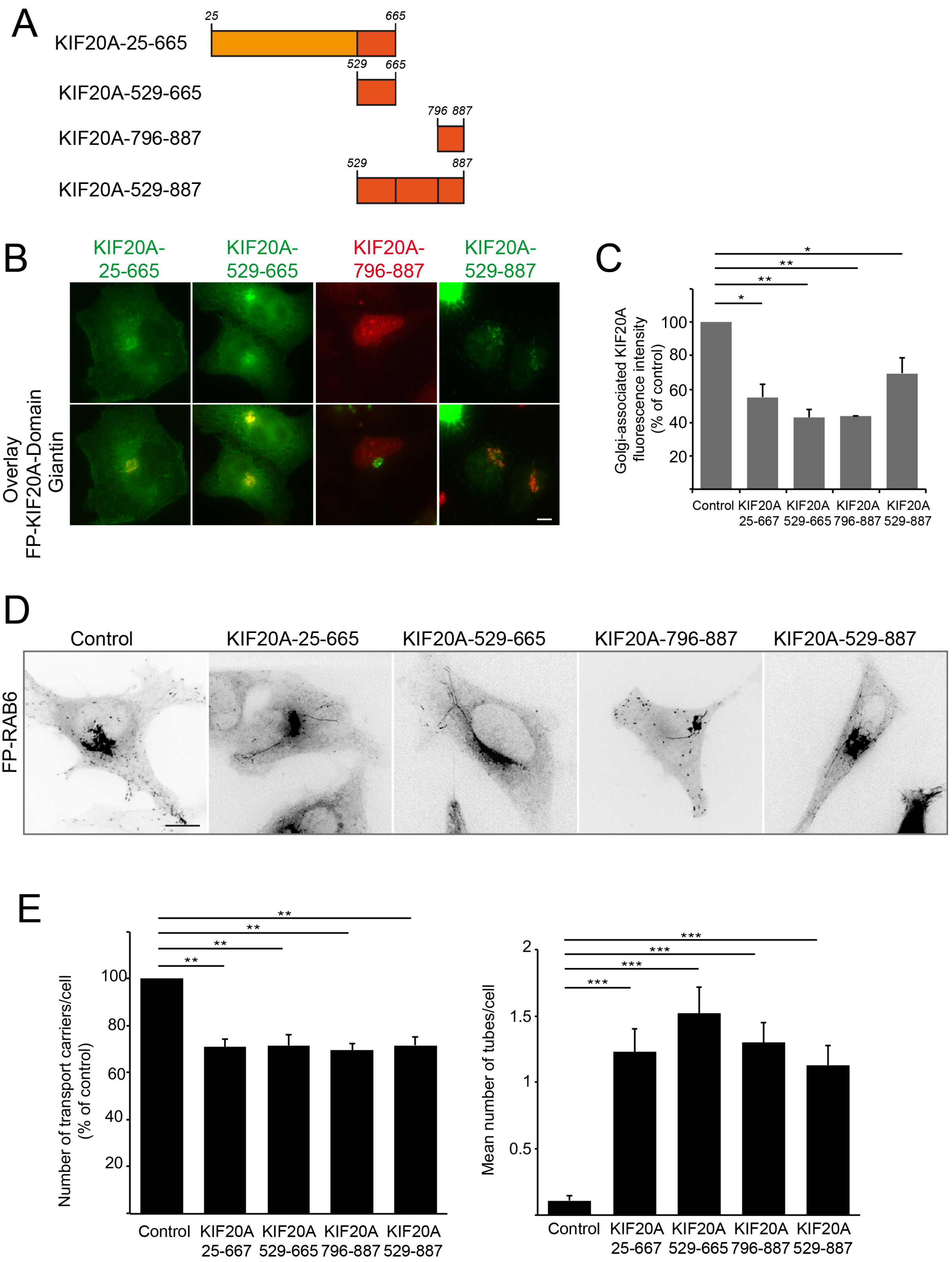
A
KIF20A-25-665
25
665
529
KIF20A-529-665
KIF20A-796-887
796
887
KIF20A-529-887
B
KIF20A-25-665
KIF20A-529-665
KIF20A-796-887
KIF20A-529-887
Overlay
FP-KIF20A-Domain
Giantin
C
Golgi-associated KIF20A fluorescence intensity (% of control)
100
80
60
40
20
*
**
**
*
Control
KIF20A 25-667
KIF20A 529-665
KIF20A 796-887
KIF20A 529-887
D
Control
KIF20A-25-665
KIF20A-529-665
KIF20A-796-887
KIF20A-529-887
FP-RAB6
E
Number of transport carriers/cell (% of control)
100
80
60
40
20
**
**
**
**
Control
KIF20A 25-667
KIF20A 529-665
KIF20A 796-887
KIF20A 529-887
Mean number of tubes/cell
2
1.5
1
0.5
***
***
***
***
Control
KIF20A 25-667
KIF20A 529-665
KIF20A 796-887
KIF20A 529-887

Supplementary Figure 3: Overexpression of KIF20A dominant negative mutants leads to fission defects

(A) Scheme of different truncated versions of KIF20A. Motor domain is displayed in orange. Tail domain is displayed in red. The amino acids delimitating the different domains are indicated. **(B)** Immunostaining for endogenous Giantin in HeLa cells transfected for 24-48h with GFP-KIF20A-25-665, GFP-KIF20A-529-665, mCherry-KIF20A-796-887, GFP-KIF20A-529-887. All truncated versions are partly recruited to the Golgi complex as shown by their colocalization with Giantin. **(C)** These constructs behave as dominant negative mutants since there overexpression leads to the appearance of bi-nucleated cells (GFP-KIF20A-25-665: 28.2%; GFP-KIF20A-529-665: 11.1%; mCherry-KIF20A-796-887: 6.9%; GFP-KIF20A-529-887: 8.6%; n=58-170 cells) and to a decrease in endogenous KIF20-Golgi associated staining. Results are expressed as mean ± SEM (n=33-238 cells). * p=0.02, ** $p<2.10^{-3}$ (Student's t-test). These truncated versions behave as dominant negative mutants likely because they all contain the coiled-coil domain involved in the homodimerization of KIF20A and hence could inhibit the interaction of KIF20A with specific partners. **(D)** mCherry-RAB6 expressing HeLa cells were co-transfected with GFP-KIF20A-25-665, GFP-KIF20A-529-665, GFP-KIF20A-529-887 or GFP-RAB6 expressing HeLa cells were co-transfected with mCherry-KIF20A-796-887 and imaged by spinning-disk confocal time-lapse videomicroscopy. **(E)** Quantification of the number of transport carriers and of Golgi-connected membrane tubes after expression of the different KIF20A dominant negative mutants. Results are expressed as mean ± SEM (n= 27-59 cells). ** $p<10^{-3}$, *** $p<10^{-10}$ (Student's t-test). Bars, 10 µm.

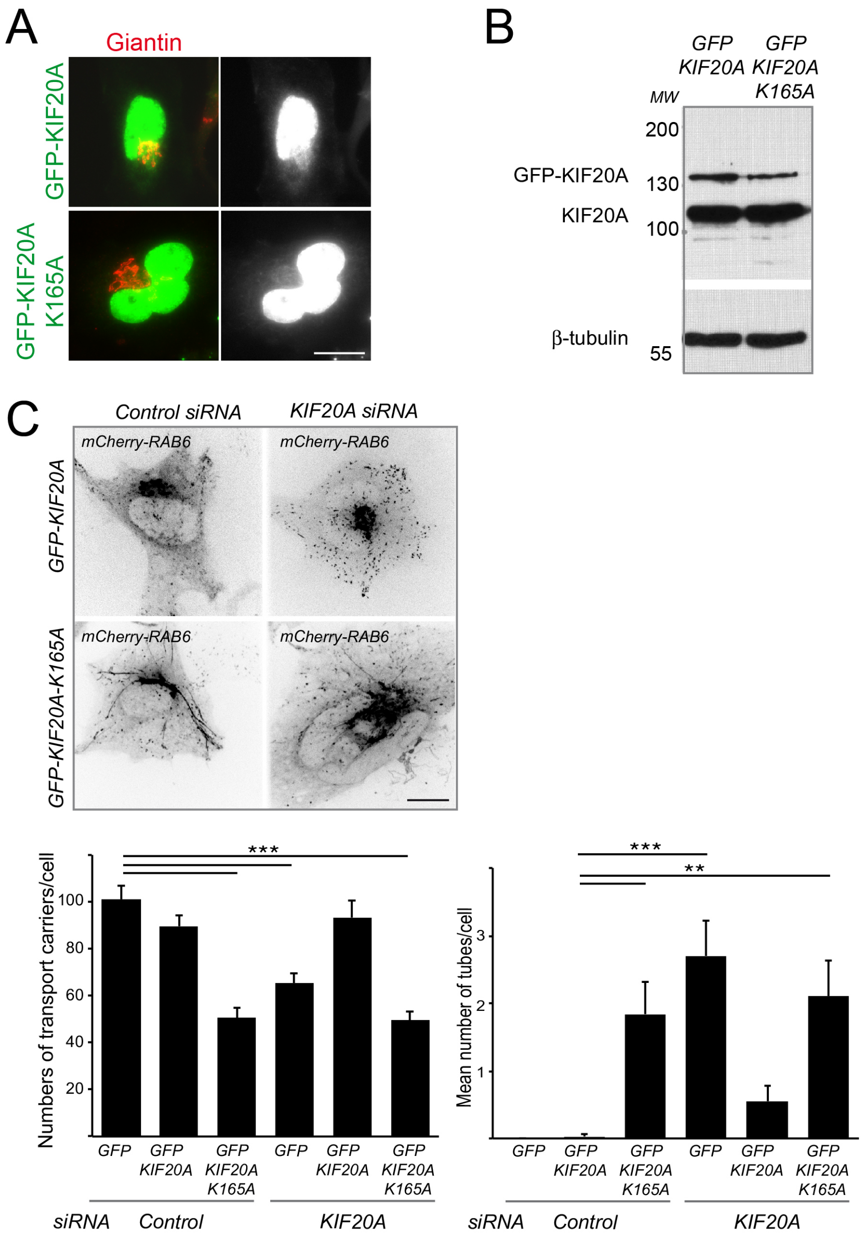

<u>Supplementary Figure 4 : The motor domain integrity of KIF20A is required for the fission process.</u>

(A) Localization of GFP-KIF20A and GFP-KIF20A-K165A overexpressed in HeLa cells. As wild-type KIF20A, a fraction of mutant KIF20A is detected in the Golgi region (labelled with Giantin). Bar, 10 µm. **(B)** Western-blot analysis of HeLa cells transfected for 24h with GFP-KIF20A or GFP-KIF20A-K165A. GFP-KIF20A and GFP-KIF20A-K165A relative expression was revealed either with an anti-KIF20A antibody. β-tubulin signal was used as a loading control. **(C)** mCherry-RAB6 and GFP-KIF20A or GFP-KIF20A-K165A were expressed in control or KIF20A siRNA transfected cells. Only mCherry-RAB6 signal is shown. Bottom: Quantification of the number of transport carriers and of Golgi-connected membrane tubes after expression of the different constructs. Results are expressed as mean ± SEM (n= 18-28 cells). ** $p<10^{-3}$, *** $p<10^{-5}$ (Student's t-test). Bar, 10 µm.

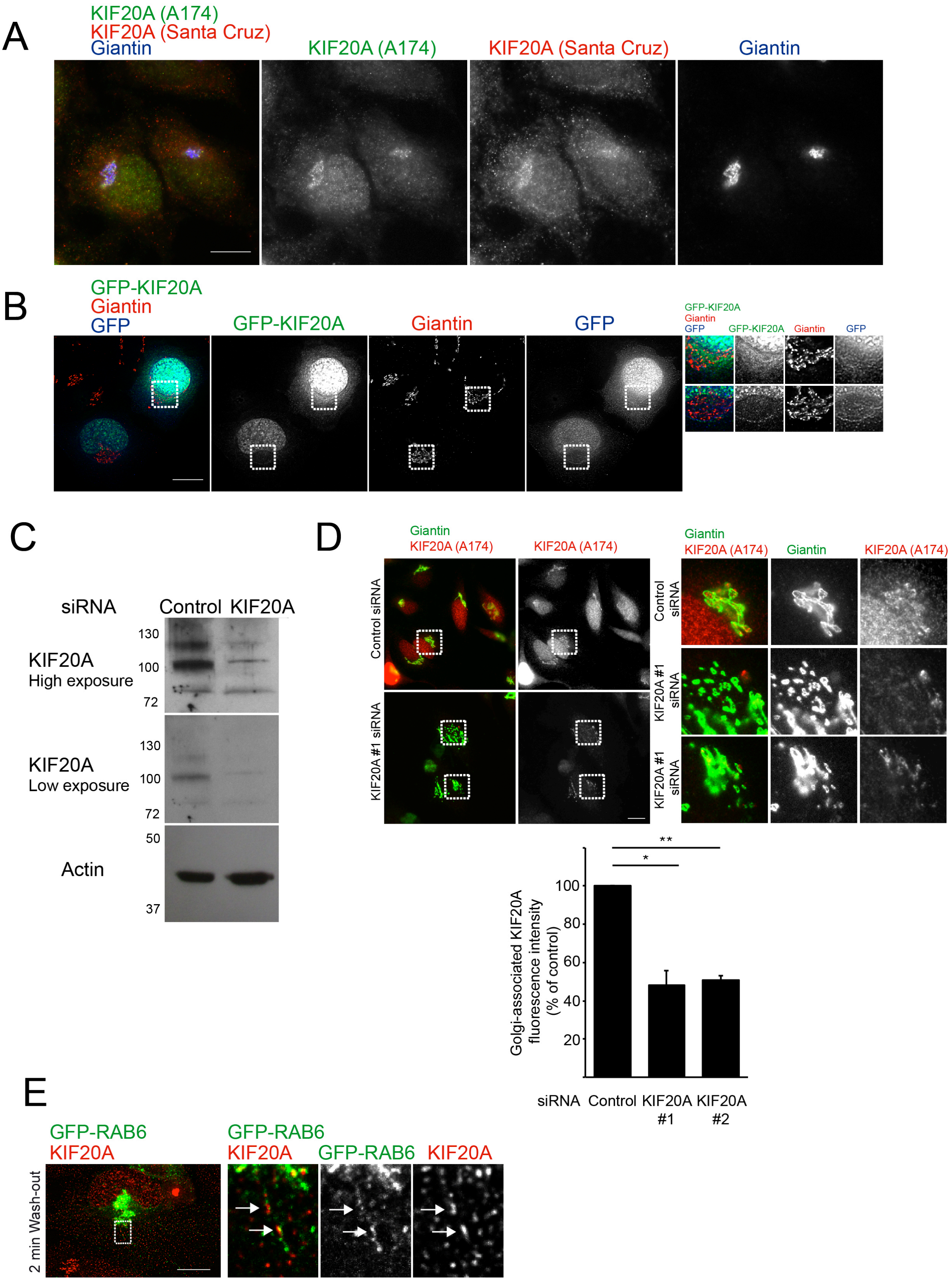
A
KIF20A (A174)
KIF20A (Santa Cruz)
Giantin
KIF20A (A174)
KIF20A (Santa Cruz)
Giantin
B
GFP-KIF20A
Giantin
GFP
GFP-KIF20A
Giantin
GFP
GFP-KIF20A
Giantin
GFP
GFP-KIF20A
Giantin
GFP
C
siRNA
Control
KIF20A
KIF20A
High exposure
130
100
72
KIF20A
Low exposure
130
100
72
Actin
50
37
D
Giantin
KIF20A (A174)
KIF20A (A174)
Control siRNA
KIF20A #1 siRNA
Giantin
KIF20A (A174)
Giantin
KIF20A (A174)
Control siRNA
KIF20A #1 siRNA
KIF20A #1 siRNA
Golgi-associated KIF20A fluorescence intensity (% of control)
100
80
60
40
20
**
*
siRNA
Control
KIF20A #1
KIF20A #2
E
GFP-RAB6
KIF20A
2 min Wash-out
GFP-RAB6
KIF20A
GFP-RAB6
KIF20A

Supplementary Figure 5: Specificity of the KIF20A Golgi localization. KIF20A is found at sites where fission occurs.

(A) Staining of KIF20A using an antibody described in [5], named A174, (green), an antibody from Santa Cruz (red) and Giantin (blue). **(B)** Staining of GFP-KIF20A (KIF20A-BAC, a kind gift from A. Hyman, Dresden, Germany), Giantin (red) and GFP (blue) with an antibody against GFP (blue). KIF20A is upregulated during mitosis and localizes to the nucleus in G2 phase [3]. GFP-KIF20A staining in the nucleus thus corresponds to cells in G2 phase. **(C)** Western-blot analysis of HeLa cells treated for 3 days with control or KIF20A siRNAs. KIF20A was revealed with the A174 antibody. Actin signal was used as a loading control. **(D)** Left panel: Immunostaining for endogenous Giantin and KIF20A (A174) in HeLa cells treated for 3 days with control and KIF20A siRNAs (higher magnifications of the Golgi are shown on the right). Bottom panel: Quantification of the Golgi-associated KIF20A fluorescence intensity in HeLa cells treated for 3 days with control and two different KIF20A siRNAs. Results are expressed as mean ± SEM (n= 25-32 cells). * p= 0.02, ** p= 0.002 (Student's t-test). Bars, 10 µm. **(E)** KIF20A is found at sites where fission occurs. GFP-RAB6 cells were treated for 40 min with Blebblistatin, then the drug was washed-out for 2 min. Staining of GFP-RAB6 and endogenous KIF20A (red). Higher magnifications of the fission sites along a RAB6-positive tube are shown on the right. Arrows point at sites of fission where GFP-RAB6 and KIF20A are found accumulated and colocalized. These sites correspond to sites where fission occurs [6]. Bars, 10 µm.

A

	RAB6 Q72L	KIF20A-1-530	KIF20A-1-665	KIF20A-529-887	KIF20A-529-665	KIF20A-761-887
MyoIIA-1147-1653	+	n.d.	n.d.	n.d.	+	n.d.
MyoIIA-1248-1374	+	-	+	+	+	+
MyoIIA-1248-1328	+	-	+	+	+	+
MyoIIA-1329-1471	+	-	+	+	+	+

B

	RAB6 Q72L	KIF20A-529-665	MyoIIA-1147-1653
KIF20A-529-665	+	+	+
KIF20A-529-665 K629W-S631W	-	+	+
KIF20A-529-665 K629W	-	+	+
KIF20A-529-665 S631W	-	+	+
KIF20A-529-665 L628W	+	+	+

C

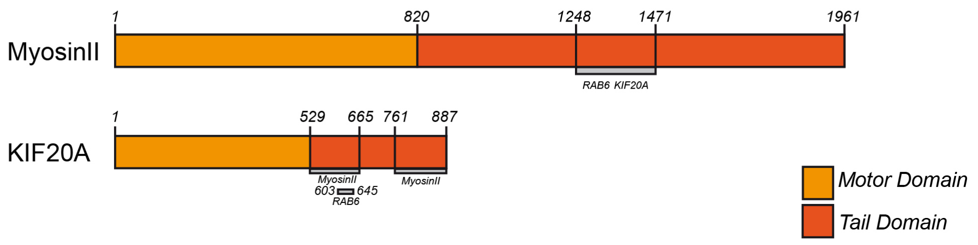

Supplementary Figure 6: Mapping of the binding domains of RAB6, KIF20A and Myosin IIA.

(A) Table of the interactions found using yeast two-hybrid experiments (positive or negative) between RAB6Q72L, and different fragments of KIF20A and Myosin IIA. Some of the interactions are shown in Fig. 3. (**B)** Table of the interactions found using yeast two-hybrid experiments (positive or negative) between RAB6Q72L, KIF20A 529-665 and Myosin IIA 1147-1653 fragments and mutants of KIF20A. Some of the interactions are shown in Fig. 3. (**C)** Scheme of Myosin IIA and KIF20A, showing the identified interactions domains of RAB6, KIF20A and Myosin II. Motor domain is displayed in orange. Tail domain is displayed in red. The amino acids delimitating the different domains are indicated.

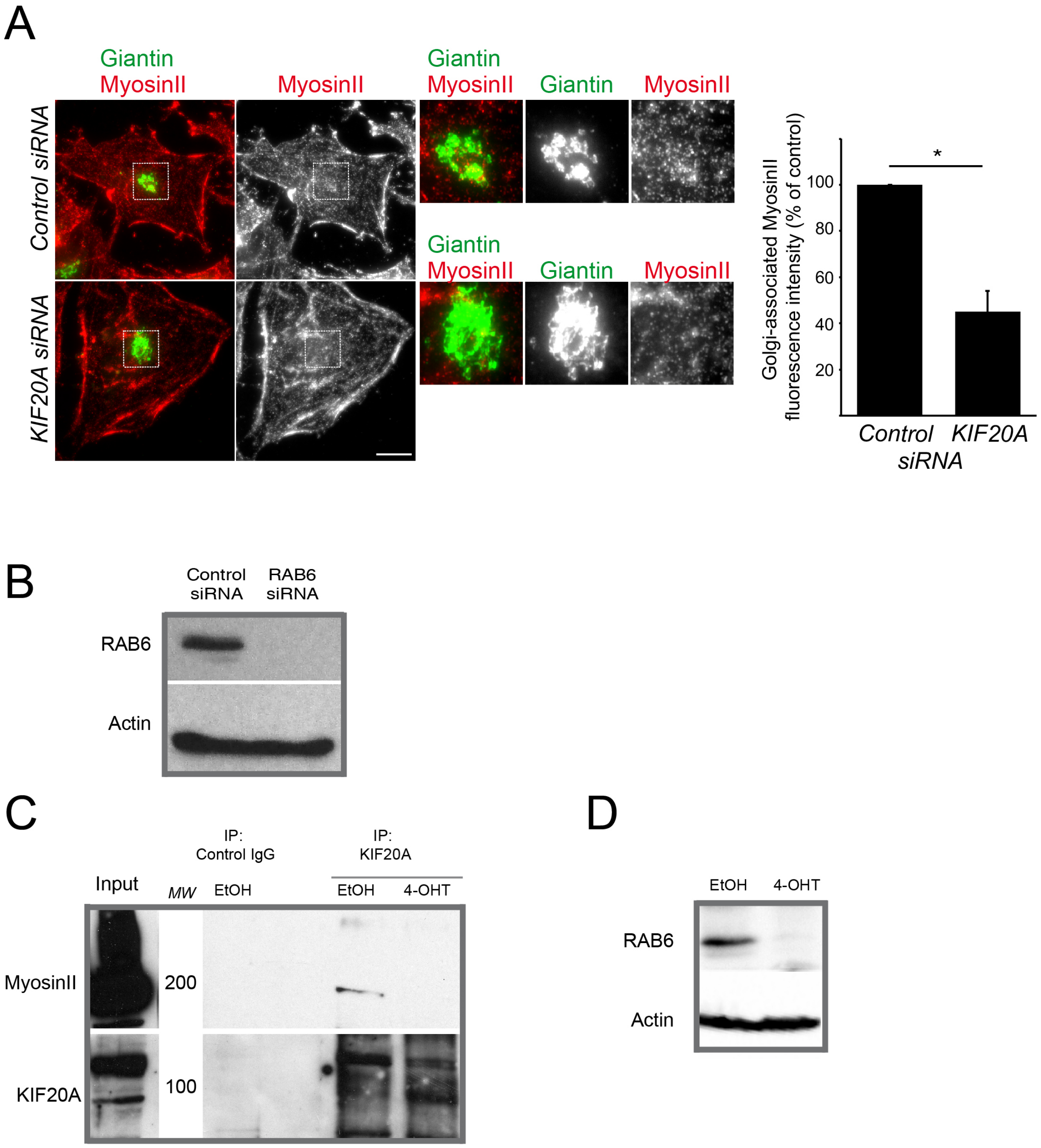
A
Giantin
MyosinII
MyosinII
Giantin
MyosinII
Giantin
MyosinII
Control siRNA
KIF20A siRNA
Giantin
MyosinII
Giantin
MyosinII
Golgi-associated MyosinII fluorescence intensity (% of control)
100
80
60
40
20
*
Control siRNA
KIF20A
B
Control siRNA
RAB6 siRNA
RAB6
Actin
C
IP: Control IgG
IP: KIF20A
Input
MW
EtOH
EtOH
4-OHT
MyosinII
200
KIF20A
100
D
EtOH
4-OHT
RAB6
Actin

Supplementary Figure 7: KIF20A stabilizes Myosin II at the Golgi complex in HeLa cells. Efficiency of RAB6 siRNA. Co-immunoprecipitation between endogenous RAB6, KIF20A and Myosin II in MEF cells.

(A) Staining for endogenous Giantin (green) and endogenous Myosin Light Chain (red) in HeLa cells 3 days after transfection with specific KIF20A siRNAs (see higher magnifications (boxes) on the right). Bar, 10 µm. Of note, KIF20A is required for cytokinesis and KIF20A depletion leads to binucleated cells [2,3]. Quantification of Golgi-associated Myosin Light Chain fluorescence intensity in cells treated as described above (mean ± SEM, n= 23-66 cells). * p= 0.01 (Student's t-test). Bar, 20 µm. **(B)** Western-blot analysis of HeLa cells treated for 3 days with control or RAB6 siRNAs. RAB6 was revealed using anti-RAB6 antibody. Actin signal was used as a loading control. **(C)** Mouse embryonic fibroblasts (MEFs) prepared from RAB6 loxP/Ko Rosa26CreERT2-TG embryos (described in [7]) were treated with EtOH (control) or 4-OHT for 96 h. Cell extracts were immunoprecipitated with control IgG or anti-KIF20A antibodies. Myosin II bound to KIF20A was revealed by Western blot analysis using an anti-Myosin II antibody. KIF20A was revealed by Western blot analysis using an anti-KIF20A antibody. Input represents a 5% load of the total cell extracts used in all conditions. *MW*, molecular weight in kDa. **(D)** Western-blot showing the efficiency of RAB6 depletion after 4-OHT treatment. RAB6 was revealed using anti-RAB6 antibody. Actin signal was used as a loading control.

Supplementary Figure 8 : Uncropped scans of the blots

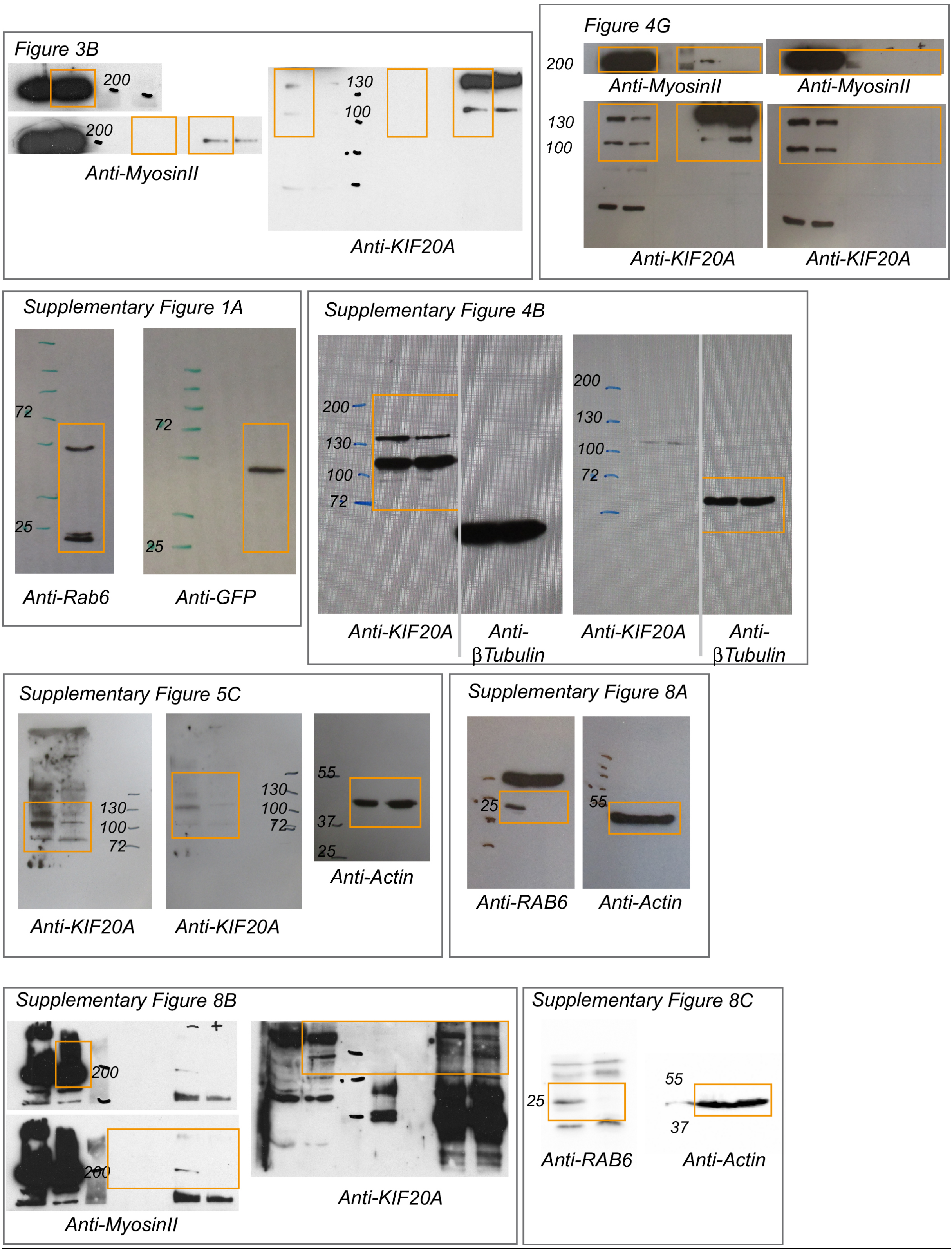

Supplementary Table 1 : Data collection and refinement statistics

	KIF20A-RBD/Rab6A
**Data collection**	
Space group	P 2_1 2_1 2
Cell dimensions	
a, *b*, *c* (Å)	125.25, 128.19, 36.03
a, b, g (°)	90, 90, 90
Resolution (Å)	44.79 - 2.088 (2.21 - 2.088)
R_{merge}	12.8(66.9)
I / sI	14.11 (2.53)
Completeness (%)	96.90 (95.7)
Redundancy	3.6 (3.6)
**Refinement**	
Resolution (Å)	44.79 - 2.088 (2.163 - 2.088)
No. reflections	34406 (3261)
R_{work} / R_{free}	0.1764 (0.2541) / 0.2042 (0.2739)
No. atoms	
Protein	3402
Ligand/ion	91
Water	251
B-factors	
Protein	56.80
Ligand/ion	43.40
Water	55.40
R.m.s. deviations	
Bond lengths (Å)	0.003
Bond angles (°)	0.73

Supplementary References

1. Kouranti, I., Sachse, M., Arouche, N., Goud, B. & Echard, A. Rab35 Regulates an Endocytic Recycling Pathway Essential for the Terminal Steps of Cytokinesis. *Current Biology* **16,** 1719–1725 (2006).
2. Hill, E., Clarke, M. & Barr, F. A. The Rab6-binding kinesin, Rab6-KIFL, is required for cytokinesis. *EMBO J.* **19,** 5711–5719 (2000).
3. Fontijn, R. D. *et al.* The human kinesin-like protein RB6K is under tight cell cycle control and is essential for cytokinesis. *Molecular and Cellular Biology* **21,** 2944–2955 (2001).
4. Guillou C., Rouleau J., Rivollier J. Talapatra S.K., Kozielski F. Carniato D., Bougeret C. New highly potent KIF20A/MKLP2 inhibitors in the paprotrain series : design, synthesis and structural-biological evaluations. *Manuscript in preparation*
5. Echard, A. Interaction of a Golgi-Associated Kinesin-Like Protein with Rab6. *Science* **279,** 580–585 (1998).
6. Miserey-Lenkei, S. *et al.* Rab and actomyosin-dependent fission of transport vesicles at the Golgi complex. *Nature Cell Biology* **12,** 645–654 (2010).
7. Bardin, S. *et al.* Phenotypic characterisation of RAB6A knockout mouse embryonic fibroblasts. *Biol. Cell* **107,** 427–439 (2015).
