# Supplementary material for: Coupling fission and exit of RAB6 vesicles at Golgi hotspots through kinesin-myosin interactions
Source: Nat Commun. 2017 Nov 1;8:1254. doi: 10.1038/s41467-017-01266-0 (PMC5665954; doi:10.1038/s41467-017-01266-0)
Supplement: Supplementary file 3 — Description of Additional Supplementary Files [file 41467_2017_1266_MOESM3_ESM.pdf]

Description of Additional Supplementary Files

File Name: Supplementary Movie 1

Description: RAB6-positive vesicles exit the Golgi complex at fission hotspots

HeLa cells expressing GFP-Rab6 were imaged using a spinning-disk microscope for 1 min at 1 sec interval. On top, still image of the Golgi with orange circles showing the location of 5 fission hotspots.

File Name: Supplementary Movie 2

Description: Golgi fission hotspots are stable in time

Movies performed on the same Golgi complex of a GFP-RAB6 expressing cell at 10 min movie interval. Left movie, t0; right movie, t+10 min. Cells were imaged using a spinningdisk microscope for 1 min at 1 sec interval. On top, still image of the Golgi with orange circles showing the location of 7 fission hotspots.

File Name: Supplementary Movie 3

Description: Golgi fission hotspots are similar to the ones where the membrane tubes are formed after inhibition of the fission process

HeLa cells expressing GFP-Rab6 were treated for 45 min with 25 µM para-nitroblebbistatin. Then para-nitro-blebbistatin was washed out. After recovery of normal GFPRAB6 vesicles trafficking, the cells were imaged using a spinning-disk microscope for 60 sec at 1 sec interval. On top, still image of the Golgi with orange lines showing the location of the fission hotspots.

File Name: Supplementary Movie 4

Description: Inhibition of KIF20A function by paprotrain inhibits the fission of Rab6-positive transport carriers from the Golgi.

HeLa cells expressing GFP-Rab6 were imaged before (left movie) or 40 min (right movie) after treatment with paprotrain (50 µM). Images were acquired using a spinning-disk microscope for 10 sec at 1 sec interval.

File Name: Supplementary Movie 5

Description: Inhibition of KIF20A function by paprotrain affects the diffusion of GFP-RAB6 on Golgi membranes.

HeLa cells expressing GFP-RAB6 were bleached in a 30 pixels circular region of the Golgi apparatus (white circle) and then imaged for 2 min at 1sec interval using a spinning disk microscope in control cell (left movie) or in a cell treated for 30 min with paprotrain (right movie).
